# *Toxoplasma gondii* infection affects the complete blood count and disturbs the markers of oxidative stress from the vital organs of wild rodents

**DOI:** 10.1038/s41598-024-73265-3

**Published:** 2024-09-30

**Authors:** Maryam Ijaz, Asmat Ullah Khan, Shakir Ullah, Afshan Khan, Samir Ibenmoussa, Baye Sitotaw, Turki M. Dawoud, Adil Khan, Furhan Iqbal

**Affiliations:** 1 Institute of Zoology, Bahauddin Zakariya University, Multan, 60800 Pakistan; 2 Department of Zoology, Shaheed Benazir Bhutto University, Sheringal, Dir Upper, Khyber, Pakhtunkhwa Pakistan; 3 Department of Zoology, Abdul Wali Khan University, Mardan, Pakistan; 4 Laboratory of Therapeutic and Organic Chemistry, Faculty of Pharmacy, University of Montpellier, 34000 Montpellier, France; 5 Department of Biology, Bahir Dar University, P.O. Box 79, Bahir Dar, Ethiopia; 6 Department of Botany and Microbiology, College of Science, King Saud University, P.O. Box 2455, Riyadh, 11451 Saudi Arabia; 7 Department of Botany and Zoology, Bacha Khan University, Charsadda, Khyber, 24420 Pakhtunkhwa Pakistan

**Keywords:** *Toxoplasma Gondii*, Molecular prevalence, Phylogeny, Pakistan, Wild rodents, Microbiology, Health occupations

## Abstract

Rodents are the synanthropic mammals that are existing in close proximity to humans and their belongings and have the potential to act as the reservoir for a variety of parasites having zoonotic potential. Present study was designed to report the molecular prevalence and phylogenetic evaluation of *Toxoplasma gondii* in the blood samples of four wild rodent species [*Rattus rattus* (*N* = 122), *Mus musculus* (*N* = 64), *Rattus norvegicus* (*N* = 57) and *Dryomys nitedula* (*N* = 1)] that were trapped during May 2022 till July 2023 from three districts in Punjab (Jampur, Dera Ghazi Khan and Multan) and three districts (Upper Dir, Mardan and Bunar) in Pakistan. Results revealed that 44/244 (18%) rodents amplified *ITS-1* gene of *Toxoplasma gondii* through PCR. Parasite prevalence varied between the rodent species. Highest rate of infection was found in *Rattus norvegicus* followed by *Rattus rattus* and *Mus musculus*. For both rat species, *Toxoplasma gondii* infection significantly varies between the sampling districts. DNA sequencing and BLAST analysis confirmed the presence of *Toxoplasma gondii* in rodent blood samples. Phylogenetic analysis showed that Pakistani isolates were genetically diverse and clustered with the isolates that were reported from worldwide countries. Complete blood count analysis revealed that parasite infected rodents had disturbed lymphocyte, mean platelet volume, mean corpuscular volume (and mean corpuscular hemoglobin concentration. Markers of oxidative stress analysis revealed that infected rodent had elevated malondialdehyde levels in liver and kidney while disturb catalase concentrations in kidney and heart as compared to uninfected animals. In conclusion, we are reporting a relatively high prevalence of *Toxoplasma gondii* in Pakistani rodents. Infection leads to disturbed complete blood count and markers of oxidative stress in the vital organs. We recommend large scale studies in various geo-climatic regions of Pakistan to report the incidence and prevalence of this pathogen among the rodents in order to prevent their infections in local people as well as in livestock.

## Introduction

Rodents are successfully combating with different environments (aquatic, semiaquatic or dry conditions) and among them synanthropic rodents are especially important as they share the common environment with human and are considered as serious reservoirs of the pathogens^1^. Among the mammals, the largest order is Rodentia that included a number of rats and mice species that not only acts as pest but they are also involved in the transmission of a number of parasitic infections including toxoplasmosis^2^.

*Toxoplasma gondii* is an intracellular protozoan that infects almost all warm-blooded animals and several cold-blooded organisms across the globe^3^. Rodents are known to have different susceptibility to various strains of this parasite. Rodents that are sensitive to a particular strain usually die soon while the resistant rodents develop lifelong chronic infection that plays a major role in the transmission of parasite^4^. The prevalence of *Toxoplasma gondii* in wildlife is correlated with the presence of final hosts (felids) as the oocysts of this parasite is excreted in the fecal material and ingested by new host^5,6^. Since the rat and mice are omnivorous and consumes seeds and insects and materials from the environment that can be contamination by *Toxoplasma gondii*’s oocysts, hence they have high susceptibility to get this parasitic infection^7^. These infected rodents are hunted and consumed by cats, dogs and other animals that carry this infection to new hosts and destinations^5^. The most common symptoms of toxoplasmosis in animals include fever, loss of appetite and lethargy but symptoms vary with the nature of infection: acute or chronic^8^.

Literature review revealed just a couple of studies regarding the *Toxoplasma gondii* mediated infection in wild rodents from Pakistan. Both studies were from Punjab province: Rizwan et al.^9^ used PCR while Ahmad et al.^10^ used ELISA to report the prevalence of this protozoan in rodent species. Hence the present study was designed to report the prevalence of *Toxoplasma gondii* in the blood samples of four wild rodent species (*Mus musculus*, *Rattus norvegicus*,* Rattus rattus* and *Dryomys nitedula*) that were trapped from six districts in two provinces (Punjab and Khyber Pakhtunkhwa) in Pakistan. We are also reporting the risk factors and effect of parasite on the complete blood count and on the markers of oxidative stress from the vital organs of these rodents.

## Materials and methods

### Study area and subjects

In the present work, all methods and experiments were approved by the ethical review committee of Bahauddin Zakariya University Multan, Pakistan (BZU/Ethics/Zool-29-2022). Importantly, all methods were performed in accordance with the relevant guidelines and regulations.

**Fig. 1:**
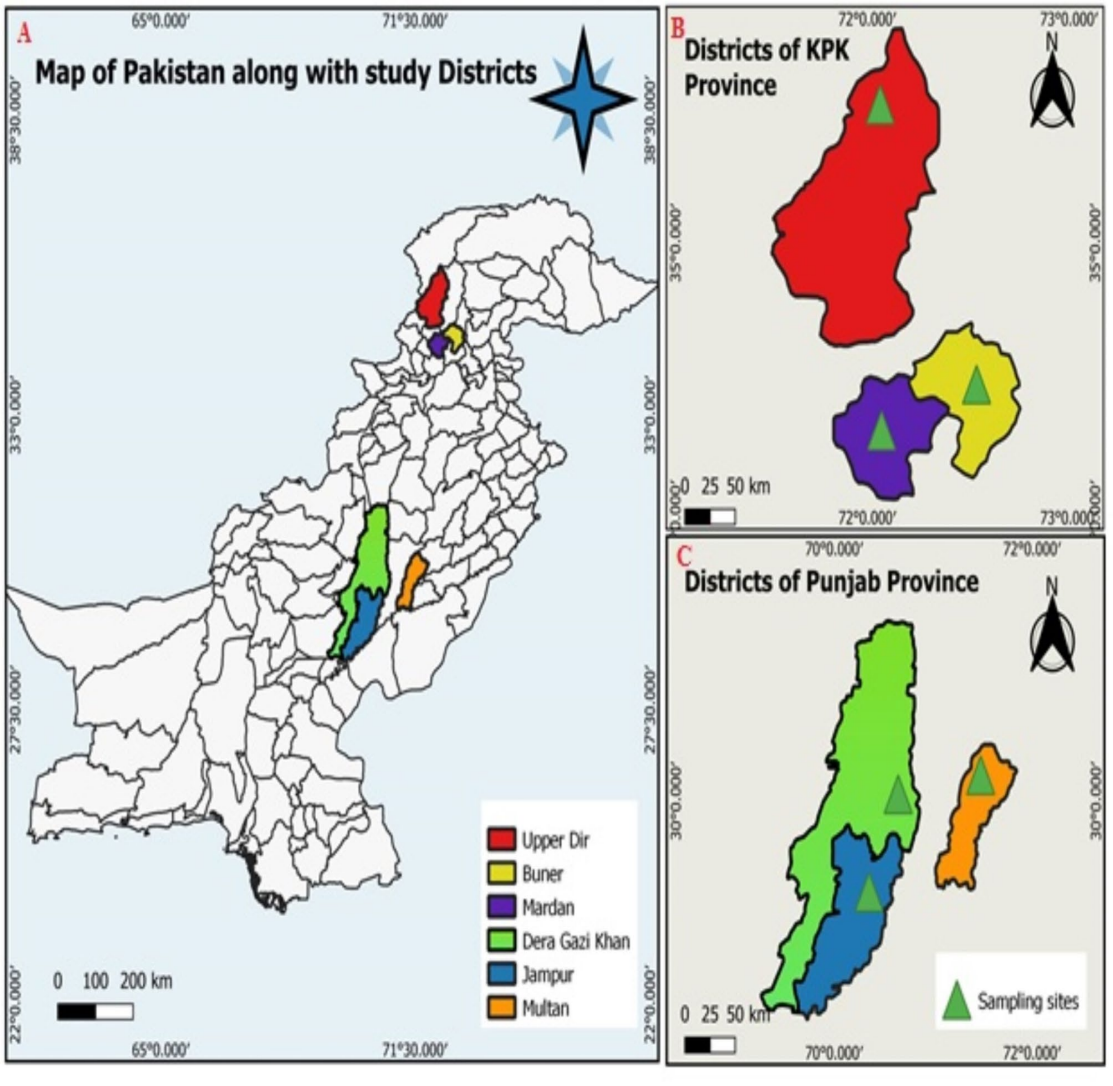
(**A**) Map of Pakistan showing the sampling sites. Magnified maps of the districts in (**B**) Khyber Pakhtunkhwa and (**C**) Punjab from where the rodent samples were collected.

An active epidemiological survey was conducted to determine the prevalence of *Toxoplasma gondii* in wild rat and mice that were trapped from three regions in Punjab (Jampur, Dera Ghazi Khan and Multan) and three regions in Khyber Pakhtunkhwa (KPK) (Sheringal, Mardan and Buner) in Pakistan (Fig. 1). A total of 244 wild rodents were trapped from two provinces during May 2022 till July 2023. Standard rodent traps with bate or glue traps were set in houses, storage sites, fields and in shops the sampling areas in Punjab. Along with the urban regions, traps were also set in forest and mountain regions in Khyber Pakhtunkhwa along with the usual sites as in Punjab province. The traps were checked at regular intervals so that live rodents can be handled for blood collection under isoflurane inhalation. Standard taxonomic keys were used to identify the species of the trapped rodents^11^.

### Data collection

A questionnaire was filled for each animal on sampling site to gather basic information about each trapped rodent including species, sex, sampling site, body weight and length and presence of ectoparasites on animal’s body.

### Blood collection, complete blood count and DNA extraction

Blood samples were collected from each animal from direct cardiac puncture. Blood samples were collected into the screw-capped tubes containing 0.5 M Ethylene Diamine Tetra Acetic acid (EDTA) as an anticoagulant and frozen at − 20 °C till the further molecular analysis. Hematological parameters, red, white blood cell and platelet counting and associated parameters were analyzed in all collected rodent blood samples by using an automated hematological analyzer (Mythic™ 18 Vet, Orphee, Switzerland). Genomic DNA was extracted from the rodent blood by using Wizard^®^ Genomic DNA Purification Kit (Promega, Madison, WI, USA) following the manufacturer’s instructions.

### Molecular detection by PCR

The extracted DNA samples were analyzed for the presence of *Toxoplasma gondii* (target was *ITS-1* gene) by using the species-specific primers (Forward 5′-AGTTTAGGAAGCAATCTGAAAGCACATC-3′ and Reverse 5′-GATTTGCATTCAAGAAGCGTGATAGTAT-3′) following Halova et al.^12^. A reaction mixture of 25 µl was prepared that contained 13 mM Tris–HCl (pH 8.3), 65 mM KCl, 2.5 mM MgCl_2_, 300 µM of each dNTP,1U of DNA Polymerase, 0.5µM of each primer and 5 µl of template DNA. Reaction conditions comprised an initial denaturation step of 94 °C for 3 min followed by 30 cycles of denaturation 94 °C for 30 s, primer annealing 55 °C for 45 s and extension 72 °C for 30 s. A final extension at 72 °C for 5 min was performed. During each reaction, distilled water was used as negative control while DNA of *Toxoplasma gondii* (that was available at our lab from previous studies) was used as positive control.

### DNA sequencing

Amplified PCR products were sequenced by a commercial company (First Base, Malaysia). All sequences were submitted to NCBI’s GenBank and were assigned accession numbers: OR797081, OR797082, OR797083 and OR797084 for *Toxoplasma gondii.*

### Phylogenetic analysis of ***ITS-1*** gene of ***Toxoplasma gondii***

The DNA sequences generated in this study underwent initial trimming by using FinchTV (version 1.4.0) to eliminate the primer-contaminated regions and any misread nucleotides at the start and end of the sequence. Additional similar sequences were retrieved using the Basic Local Alignment Search Tool (BLAST) algorithm on NCBI’s platform. Subsequently, all sequences were aligned using the ClustalW multiple sequence alignment algorithm in BioEdit (version 7.2.5). The aligned sequences were imported into MEGA X (version 10.2.6). A model selection test was conducted for all sequences, using MEGA’s integrated model selection tool. The best-fit model was chosen based on Bayesian Information Criteria (BIC) and Akaike Information Criteria (AIC) values, with the model exhibiting the lowest BIC and AIC considered as the “best-fit” substitution model. Phylogenetic trees were constructed using the Maximum Likelihood algorithm in MEGA X with 1000 bootstraps. The final version of the inferred tree was generated using the iTOL server (https://itol.embl.de/, accessed on November 1st, 2023).

### Oxidative stress marker analysis

Animals were sacrifices under Isoflurane anesthesia and vital organs (heart, kidney, lungs and liver) were surgically removed and markers of oxidative stress: Superoxide dismutase, Malondialdehyde and Catalase levels were determined in them following Hussain et al.^13^.

### Statistical analysis

Statistical package Minitab (Minitab, Pennsylvania, USA) was used for the statistical analysis of data. Data was expressed as mean values ± standard error of mean (SEM) where applicable. Probability levels of *P* < 0.05 were considered significant. PCR based pathogen prevalence between various rodents’ species was calculated by Chi-square (χ^2^) test. One way ANOVA was applied to analyze the prevalence of a parasite between sampling sites, Association between the presence of pathogen and studied epidemiological factor was assessed by contingency table analysis using the Fisher’s exact test (for 2 × 2 tables). Two sample t-test was calculated to compare hematological between parasite positive and negative rodents.

## Results

### Subject details

From Punjab, 40, 48 and 36 rodents were trapped from Jampur, Dera Ghazi Khan and Multan districts respectively belonging to three species: *Mus musculus*, *Rattus rattus* and *Rattus norvegicus*. While 58, 34 and 27 rodents were captured from Upper Dir, Mardan and Buner districts in Khyber Pakhtunkhwa respectively belonging to four species: *Mus musculus*, *Rattus rattus*, *Rattus norvegicus* and *Dryomys nitedula*. The rodents included in this study were 57% male (139/244) and 43% females (105/244).

### Prevalence of ***Toxoplasma gondii*** in rodents blood samples

Polymerase chain reaction had amplified a 300 base pairs amplicon specific for *ITS-1* gene of *Toxoplasma gondii* in 44 out of 244 (18%) blood samples that were collected from four wild rodent species during present study (Table 1).

**Table 1:** Over all comparison of *Toxoplasma Gondii* prevalence among the four rodent species captured from six sampling districts during present investigation.

Rodent species	Toxoplasma gondii positive (%)	Toxoplasma gondii negative (%)	Chi square value	*P* value
*Mus musculus*	7/64 (10.9%)	57/64 (89.1%)		
*Rattus rattus*	15/122 (12.3%)	107/122 (87.7%)		
*Rattus norvegicus*	21/57 (36.8%)	36/57 (63.2%)	24.432	*P* < 0.001***
*Dryomys nitedula*	1/1 (100%)	0/1 (0%)		
**Total**	**44/244 (18%)**	**202/244 (82%)**		

*P* < 0.001 = Highly significant (***).

% prevalence is given in parenthesis. P- value represents the output of Chi square test.

### Genetic diversity of ***Toxoplasma gondii*** in rodents

**Fig. 2:**
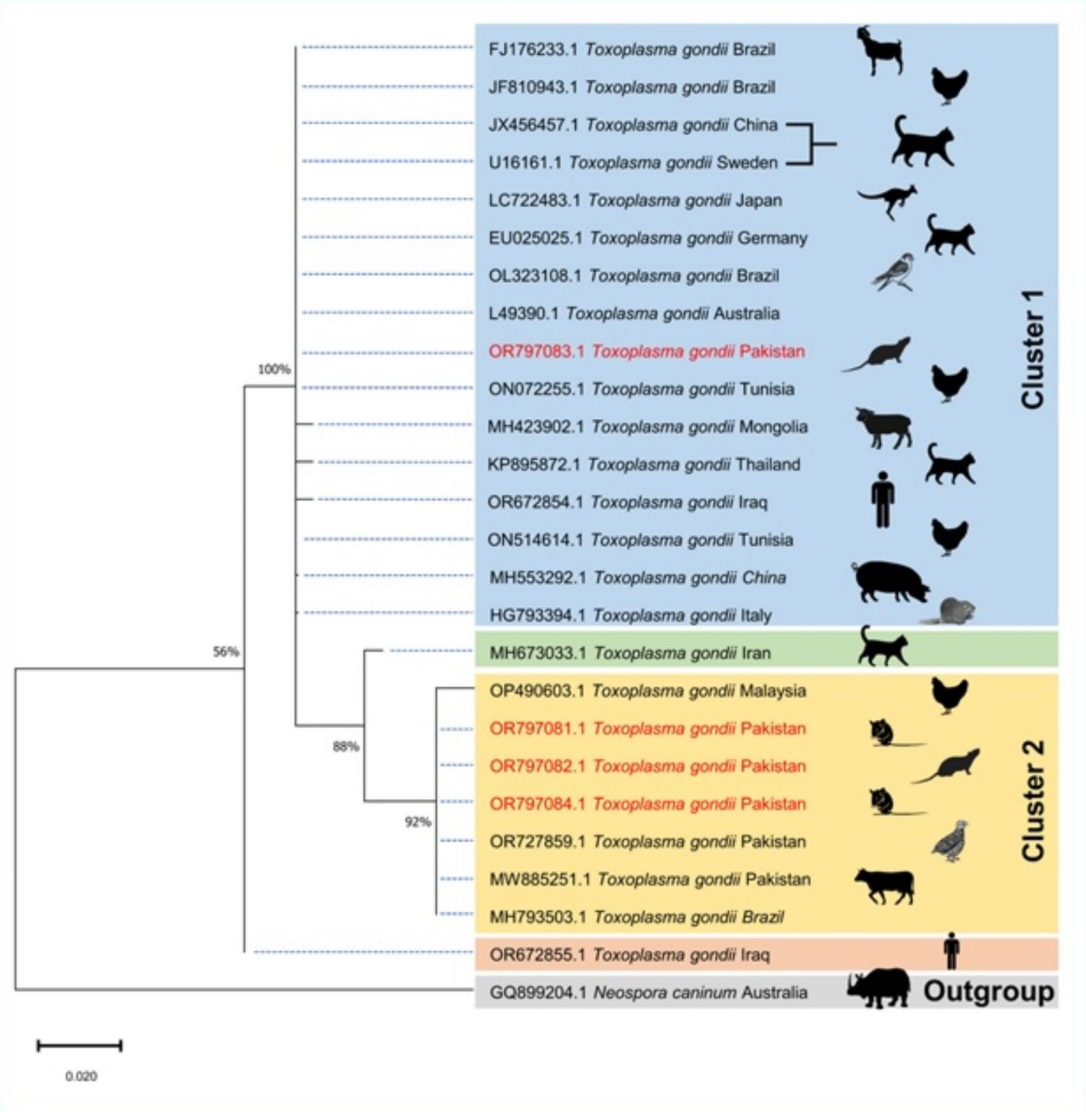
Phylogenetic tree of *Toxoplasma gondii* based on the partial *ITS-1*gene sequences. The four new sequences of *Toxoplasma gondii* obtained in this study are highlighted in red (OR797081-OR797084). Scale bar represents 0.02 substitutions per nucleotide position. Bootstrap value is shown as number on the node. The evolutionary history was inferred by using the Maximum Likelihood method and Kimura 2-parameter model^52^. The tree with the highest log likelihood (− 511.25) is shown. The percentage of trees in which the associated taxa clustered together is shown next to the branches. Initial tree(s) for the heuristic search were obtained automatically by applying Neighbor-Join and BioNJ algorithms to a matrix of pairwise distances estimated using the Maximum Composite Likelihood (MCL) approach, and then selecting the topology with superior log likelihood value. The tree is drawn to scale, with branch lengths measured in the number of substitutions per site. The proportion of sites where at least 1 unambiguous base is present in at least 1 sequence for each descendent clade is shown next to each internal node in the tree. This analysis involved 26 nucleotide sequences. There was a total of 242 positions in the final dataset. Evolutionary analyses were conducted in MEGA X ^53^.

The phylogenetic tree was inferred based on these four partial *ITS-1* gene sequences by Maximum-Likelihood method. The label in the tree includes the accession number, name of the parasite and the country from where this haplotype was deposited. Host of *Toxoplasma gondii* is also shown as graphic image. The haplotypes generated in this study displayed genetic diversity as three of them clustered together while one: ‘*Toxoplasma gondii* OR797083.1 (isolated from *Rattus rattus*) Pakistan’, clustered with *Toxoplasma gondii ITS-1* sequences reported from Brazil (FJ176233, JF810943, OL323108), China (JX456457, MH553292), Sweden (U16161), Japan (LC722483), Australia (L49390), Germany (EU025025), Italy (HG793394), Tunisia (ON072255, ON514614), Iraq (OR672854), Mongolia (MH423902), and Thailand (KP895872). Whereas, the other three haplotypes: *Toxoplasma gondii* OR797081.1 (isolated from *Mus musculus*), OR797082.1 (isolated from *Rattus rattus*) and OR797084.1 (isolated from *Rattus norvegicus*)’ generated during the present study clustered together with *Toxoplasma gondii* sequences from Pakistan (OR727859.1, MW885251.1), Malaysia (OP490603.1) and Brazil (MH793503.1). The partial *ITS-1* sequence of *Neospora caninum*’s (GQ899204) isolated from aborted fetus of Western Black Rhinoceros of Australia was used as an outgroup in this phylogenetic analysis (Fig. 2).

### Risk factor analysis

Four rodent species were identified during present investigation. Chi square test results indicated that the prevalence of *Toxoplasma gondii* significantly varied among the enrolled rodent species (*P* < 0.001). *Dryomys nitedula* was most highly susceptible to this haemoparasite infection (100%) followed by *Rattus norvegicus* (36.8%), *Rattus rattus* (12.3%) and *Mus musculus* (10.9%) (Table 1).

Rodent blood samples were collected from six different districts in two provinces during present study. When the prevalence of *Toxoplasma gondii* was compared among the sampling site, it was observed that prevalence of *Toxoplasma gondii* varied non significantly between the sampling sites (*P* = 0.2). A significant variation in *Toxoplasma gondii* prevalence with sampling sites was observed for *Rattus rattus* (*P* < 0.001). The highest parasite prevalence was observed in rats trapped from Jampur (100%) followed by Buner (31%), Upper Dir (19%), Multan (4%) and Dera Ghazi Khan District (3%). None of the *Rattus rattus* trapped from Mardan district was *Toxoplasma gondii* infected. Prevalence of *Toxoplasma gondii* in *Rattus norvegicus* also varied significantly between the sampling sites (*P* < 0.001). The highest parasite prevalence was observed in *Rattus norvegicus* captured from Mardan (70%) followed by Buner (67%), Jampur (50%), Dera Ghazi Khan (50%) and Multan (8%) district. *Toxoplasma gondii infection* was not detected in *Rattus norvegicus* captured from Upper Dir District (Table 2).

**Table 2:** Over all comparison of *Toxoplasma Gondii* prevalence among rodents of a particular species captured from six sampling districts during present investigation.

Sampling district	Infected Mus musculus	Uninfected Mus musculus	*P* value	Infected Rattus rattus	Uninfected Rattus rattus	*P* value	Infected Rattus norvegicus	Uninfected Rattus norvegicus	*P* value
Jampur *N* = 40	3/34 (10%)	31/34 (90%)		2/2 (100%)	0/2 (0%)		2/4 (50%)	2/4 (50%)	
Dera Ghazi Khan *N* = 48	–	–		1/30 (3%)	29/30 (97%)		9/18 (50%)	9/18 (50%)	
Multan *N* = 36	–	–	**0.2**	1/24 (4%)	23/24 (96%)	P < 0.001***	1/12 (8%)	11/12 (92%)	P < 0.001***
Upper Dir *N* = 58	4/16 (25%)	12/16 (75%)		6/32 (19%)	26/32 (81%)		0/10 (0%)	10/10 (100%)	
Mardan *N* = 34	0/6 (0%)	6/6 (100%)		0/18 (0%)	18/18 (100)		7/10 (70%)	3/10 (30%)	
Buner *N* = 27	0/8 (0%)	8/8 (100%)		5/16 (31%)	11/16 (69%)		2/3 (67%)	1/3 (33%)	

N represents the total rodents captured from a sampling district. % prevalence is given in parenthesis. P-value represents the output of one- way ANOVA test.

*P* < 0.05 = non significant; *P* < 0.001 = highly significant (***).

Significant values are given in bold.

Association of rodent sex was also determined with the prevalence of *Toxoplasma gondii*. Fisher’s exact test results indicated that *Toxoplasma gondii* infection was not associated with the sex or either *Mus musculus*,* Rattus rattus* or *Rattus norvegicus*. This analysis was not possible for *Dryomys nitedula* as just a single male animal of this species was trapped during this study (Table 3).

**Table 3:** Over all association of animal sex with the prevalence of *Toxoplasma Gondii* in a particular wild rodent species captured from six districts during present study.

Rodent species	Parameters		Toxoplasma gondii positive samples	Toxoplasma gondii negative samples	*P*-value
*Mus musculus*	Sex	Male	6/37 (16%)	31/37 (84%)	0.2
		Female	1/27 (4%)	26/27 (96%)	
*Rattus rattus*	Sex	Male	9/69 (13%)	60/69 (87%)	1
		Female	6/53 (11%)	47/53 (89%)	
*Rattus norvegicus*	Sex	Male	12/32 (38%)	20/32 (62%)	0.2
		Female	8/25 (32%)	17/25 (68%)	
*Dryomys nitedula*	Sex	Male	1/1 (100%)	0/1 (0%)	#
		Female	0/0 (0%)	0/0 (0%)	

Percentage prevalence is presented in the parenthesis. P-value represents the outcome of Fischer’s exact test.

*P* > 0.05 = non significant.

^#^Statistical analysis was not possible.

### Complete blood count analysis

Analysis of complete blood cell count indicated that *Toxoplasma gondii* infected *Mus musculus* had elevated lymphocyte count (*P* = 0.05) and mean platelet volume (*P* = 0.03) while decreased mean corpuscular volume (*P* = 0.05) and mean corpuscular hemoglobin concentration (*P* < 0.001) as compared to mice where *Toxoplasma gondii* infection was not detected (Table 4).

**Table 4:** Comparison of studied complete blood count parameters between *Toxoplasma Gondii* infected and uninfected wild *rodent* species captured from various sampling sites in Pakistan.

Studied parameters	Toxoplasma gondii positive Mus musculus	Toxoplasma gondii negative Mus musculus	Toxoplasma gondii positive Rattus norvegicus	Toxoplasma gondii negative Rattus norvegicus	Toxoplasma gondii positive Rattus rattus	Toxoplasma gondii negative Rattus rattus
White blood cells ×10^3^/µl	4.09 ± 1.3	5.9 ± 0.66	5.78 ± 1.4	7.74 ± 2.4	4.35 ± 1.7	3.88 ± 1. 5
Lymphocytes ×10^3^/µl	4.32 ± 0.6	1.59 ± 0.86*	3.95 ± 0.6	6.05 ± 1.5	29.9 ± 12	20.2 ± 8
Lymphocytes (%)	40.9 ± 13	71.5 ± 2.3	70.1 ± 6.9	80.1 ± 6.3	31.5 ± 12	20.1 ± 10
Red blood cells ×10^6^/µl	5.11 ± 1.1	5.24 ± 0.18	5.78 ± 1.0	5.6 ± 1.1	3.25 ± 0.95	2.27 ± 0.87
Hemoglobin (g/dl)	12.07 ± 0.9	12.85 ± 0.35	12.55 ± 2.4	11.65 ± 1.7	6.89 ± 1.8	7.71 ± 1.5
Hematocrit (%)	33.0 ± 6.9	30.12 ± 1.2	36.5 ± 8.7	34.5 ± 6.8	20.0 ± 5.4	21.7 ± 4.5
Mean corpuscular volume (µm^3^)	56.86 ± 0.98	65.2 ± 2.1*	67.5 ± 0.5	61.5 ± 0.5**	78.1 ± 9.8	64.9 ± 10
Mean cell hemoglobin (pg)	25.67 ± 4.9	24.99 ± 0.65	21.6 ± 0.5	21.1 ± 0.5	16.16 ± 3.1	17.4 ± 2.2
Mean corpuscular hemoglobin concentration (g/dl)	33.37 ± 1.1	45.1 ± 1.4***	34.65 ± 1.5	34.7 ± 2.2	26.8 ± 5.1	29.6 ± 3.7
Platelets × 10^3^/µl	677 ± 354	327 ± 31	307 ± 93	428 ± 159	65.5 ± 32	50.5 ± 27
Mean platelet volume (µm^3^)	9.4 ± 0.62	5.72 ± 0.29*	9.1 ± 0.55	7.75 ± 1.2	9.57 ± 0.87	7.68 ± 0.66

All values are expressed as mean ± standard error of mean. P value indicates the results of two sample t-tests calculated for each parameter.

*P* > 0.05 = non significant; *P* < 0.05 = least significant (*); *P* < 0.01 = Significant (*); *P* < 0.001 = highly significant (***).

Analysis of complete blood cell count indicated that mean corpuscular volume (*P* = 0.001) was the only parameter that was significantly elevated in *Toxoplasma gondii* infected *Rattus norvegicus.* While all the studied parameters varied non significantly (*P* > 0.05) when compared between *Toxoplasma gondii* infected and uninfected *Rattus rattus* enrolled during present investigation (Table 4).

### Markers of oxidative stress analysis

Our results indicated that Superoxide Dismutase levels varied non significantly (*P* > 0.05) when compared in kidney, liver, heat and lungs of *Toxoplasma gondii* infected and uninfected mice (Table 5). A decrease in catalase concentration in kidney (*P* = 0.02) while elevation in heart (*P* = 0.03) was documented for *Toxoplasma gondii* infected mice as compared to uninfected mice. While catalase levels remained unaffected when compared in liver and lungs of *Toxoplasma gondii* positive and negative mice enrolled during present study (Table 5). A significant decrease in Malondialdehyde levels was observed in kidney and liver of *Toxoplasma gondii* infected mice while Malondialdehyde levels in heart and lungs remained unaffected upon comparison between *Toxoplasma gondii* infected and uninfected animals (Table 5).

**Table 5:** Comparison of indicators of oxidative stress from the vital organs of *Toxoplasma Gondii* infected and uninfected wild *Mus musculus* captured from various sampling sites in Pakistan.

Kidney			Liver			Heart			Lungs		
*T. gondii* positive samples	*T. gondii* negative samples	P-value	*T. gondii* positive samples	*T. gondii* negative samples	P-value	*T. gondii* positive samples	*T. gondii* negative samples	P-value	*T. gondii* positive samples	*T. gondii* negative samples	P-value
Superoxide dismutase (unit/g)											
9.71 ± 2.6	3.53 ± 0.49	0.1	4.2 ± 2.8	4.88 ± 0.62	0.8	2.33 ± 0.01	2.38 ± 0.27	0.8	4.6 ± 0.89	4.13 ± 0.28	0.6
Catalase (mg/dL)											
1396.9 ± 3.0	1471 ± 30	0.02*	1579 ± 85	1621 ± 55	0.7	1581.8 ± 18	1509 ± 26	0.03*	1630.3 ± 21	1657 ± 29	0.7
Malondialdehyde (picomol/g)											
40.2 ± 1.9	48.3 ± 2.0	0.01**	52.9 ± 4.0	63.1 ± 4.0	0.05*	60.5 ± 8.5	81.3 ± 6.2	0.1	56.1 ± 8.9	52.0 ± 2.8	0.7

Data is presented as mean ± standard error of mean. P values present the outcome of two sample t-tests conducted for each studied parameter.

*P* > 0.05 = Non significant; P *≤* 0.05 = Least significant (*); *P* < 0.01 = Significant (**).

Data analysis revealed that the Superoxide Dismutase levels in kidney, liver, heart and lungs varied remained unaffected (*P* > 0.05) when compared between *Toxoplasma gondii* infected and uninfected *Rattus norvegicus* trapped from various sampling sites during present study (Table 6). Catalase levels were significantly elevated in lungs (*P* = 0.01) while Malondialdehyde levels were decreased in kidney of *Toxoplasma gondii* infected *Rattus norvegicus.* While catalase and Malondialdehyde levels remained unaffected in other analyzed organs when compared between parasite positive and negative *Rattus norvegicus* during present investigation (Table 6).

**Table 6:** Comparison of indicators of oxidative stress from the vital organs of *Toxoplasma Gondii* infected and uninfected wild *Rattus norvegicus* captured from various sampling sites in Pakistan.

Kidney			Liver			Heart			Lungs		
*T. gondii* positive samples	*T. gondii* negative samples	P-value	*T. gondii* positive samples	*T. gondii* negative samples	P-value	*T. gondii* positive samples	*T. gondii* negative samples	P-value	*T. gondii* positive samples	*T. gondii* negative samples	P-value
Superoxide dismutase (unit/g)											
12.96 ± 1. 3	12.1 ± 0.44	0.5	8.03 ± 1.0	8.48 ± 0.63	0.7	10.3 ± 0.9	11.46 ± 0.95	0.4	11.42 ± 1.4	10.64 ± 1.0	0.7
Catalase (mg/dL)											
70.4 ± 14	73.3 ± 18	0.9	90.1 ± 3.4	98.6 ± 5.7	0.2	67.4 ± 4.9	61.9 ± 4.3	0.3	99.8 ± 3.2	84.6 ± 4.1	0.01**
Malondialdehyde (picomol/g)											
85.8 ± 5.6	105.6 ± 6.3	*P* < 0.001***	115.2 ± 8.4	114.4 ± 9.6	0.9	123.3 ± 10	108 ± 9.9	0.3	#	#	#

Data is presented as mean ± standard error of mean. P values present the outcome of two sample t-tests conducted for each studied parameter.

*P* > 0.05 = Non significant; P *≤* 0.01 = Significant (**); *P* < 0.001 = Highly significant (***).

^#^Analysis was not possible.

Analysis of oxidative stress marker data indicated non-significant variations (*P* > 0.05) for all the studied parameters from kidney, liver and heart when compared between *Toxoplasma gondii* infected and uninfected *Rattus rattus* trapped during present investigation (Table 7).

**Table 7:** Comparison of indicators of oxidative stress from the vital organs of *Toxoplasma Gondii* infected and uninfected wild *Rattus rattus* captured from various sampling sites in Pakistan.

Kidney			Liver			Heart		
*T. gondii* positive samples	*T. gondii* negative samples	P-value	*T. gondii* positive samples	*T. gondii* negative samples	P-value	*T. gondii* positive samples	*T. gondii* negative samples	P-value
Superoxide dismutase (unit/g)								
6.41 ± 1. 3	5.69 ± 0.37	0.6	8.0 ± 1.8	9.41 ± 0.72	0.5	11.9 ± 1.5	8.7 ± 0.83	1
Catalase (mg/dL)								
142.5 ± 6	154.1 ± 3.5	0.1	206.6 ± 8.9	202.2 ± 5.8	0.7	242.8 ± 7.5	229.4 ± 6.4	0.2
Malondialdehyde (picomol/g)								
187.4 ± 24	153. 8 ± 11	0.3	145 ± 22	175.7 ± 9.7	0.2	121.9 ± 11	134.5 ± 5.1	0.3

Data is presented as mean ± standard error of mean. P values present the outcome of two sample t-tests conducted for each studied parameter.

*P* > 0.05 = Non significant.

## Discussion

Rodents are often found living in areas close to human dwelling and they are already established carriers for number vector-borne parasites with zoonotic potential^14^. To date, only a couple of reports have been documented in literature regarding the molecular prevalence of *Toxoplasma gondii* in wild rodents from Pakistan. Hence the present study was designed to report the molecular prevalence of this pathogen in the blood samples of four rodent species that were collected from six districts in two provinces of Pakistan.

During the present study, overall 18% of the enrolled Pakistani wild rodents were found infected with *Toxoplasma gondii* (Table 1). Prior to this study, there is only one report from Pakistan where PCR was used to document in the presence of *Toxoplasma gondii* in Pakistani rodents. Recently, Rizwan et al.^8^ had captured 236 rats including *Rattus rattus* and *Rattus norvegicus* from three districts of the Sahiwal division in Punjab (Pakistan) and reported the presence of *Toxoplasma gondii* in the brain of 5.9% rodents. The only other study available in literature on this topic from Pakistan was documented by Ahmad et al.^9^ as they had screened rodents and human samples (300 each) from Lahore in Punjab by using Latex Agglutination Test and reported the presence of this parasite in 58.57% of *Rattus rattus*, 36.66% of *Mus musculus* and 11.33% of enrolled humans. This limited data from Pakistan clearly indicates large scale screening of this parasite in local rodents in order to prevent its zoonotic transmission. A number of studies from various parts of the World have reported the presence of *Toxoplasma gondii* in a variety of rodent species. Ode et al.^15^ had reported that 76% central rock rats in Nigeria were *Toxoplasma gondii* infected. Hosseini et al.^5^ had reported 56% ELISA based prevalence of this parasite in *Rattus rattus* of Iran. In another study from Iran, Nazari et al.^16^ had found 35% prevalence of *Toxoplasma gondii* in three rodent species. Elamin^17^ reported the presence of this parasite in 11% of *Rattus rattus* captured from Riyadh in Saudi Arabia. Ruffolo et al.^18^ found 8% prevalence in *Rattus rattus* and *Mus musculus* trapped from Brazil. Kalmár et al.^19^ had reported that 7.3% of enrolled animals from Romania, including 9 rodent species, were infected by this protozoan parasite. Normaznah et al.^20^ reported 5.9% infection rate of *Toxoplasma gondii* in five rodent species from Malaysia. Mikhail et al.^21^ had reported 4% prevalence rate in *Rattus norvegicus* and *Rattus rattus* captured from Egypt. Manabella Salcedo et al.^22^ found 3.6% infection rate in Mus musculus that were captured from poultry farms in Argentine. These diverse findings underscore the global prevalence of *Toxoplasma gondii* in wild rodent populations and highlight the importance of ongoing surveillance and research.

The genetic diversity of *Toxoplasma gondii* in rodents has been cited in the literature but it has never been investigated in Pakistani rodents. Hence, we have we used the *ITS-1* gene sequences of *Toxoplasma gondii* amplified during current study for the phylogenetic analysis. Nuclear internal transcribed spacer one (*ITS-1*) gene is among the common molecular targets that are in use for phylogenetic studies as its variability is relatively high and it the amplification is convenient^23^. BLAST analysis of the partial sequences confirmed that the rodents were infected with *Toxoplasma gondii* and the phylogenetic study revealed that the DNA sequence amplified during this investigation were genetically diverse as three of them (OR797081, OR797082 and OR797084) clustered together with *Toxoplasma gondii* sequences from quails (Accession number OR727859.1, unpublished data) and large ruminants (Accession number MW885251.1^24^), from Pakistan, chicken and pigs from Malaysia (Accession numbers OP490603^25^) and from rams in Brazil (Accession numbers MH793503^26^), While the fourth *Toxoplasma gondii* haplotype amplified in this study (OR797083) clustered with the *ITS-1* sequences of *Toxoplasma gondii* deposited from goats (Accession number FJ176233^27^), chicken (Accession number JF810943^28^), and wild birds (Accession number OL323108^29^), from Brazil, stray cats (Accession numbers JX456457^30^), and pork (Accession number MH553292, unpublished data) from China, dog from Sweden (Accession number U16161^31^), captive Parma Wallaby from Japan (Accession number LC722483, unpublished data), from Australia (Accession number L49390^32^), cats from Germany (Accession number EU025025^33^), rodents from Italy (Accession number HG793394^34^), chicken from Tunisia (Accession numbers ON072255 and ON514614, unpublished data), livestock milk from Mongolia (Accession number MH423902, unpublished data), cat feces from Thailand (Accession number KP895872^35^) from Iraq (Accession numbers OR672854, unpublished data) (Fig. 2). This extensive genetic diversity underscores the widespread distribution of *Toxoplasma gondii* and its potential transmission across diverse host species and geographical locations.

Analysis of risk factors indicated the prevalence of *Toxoplasma gondii* varied significantly among the analyzed rodents and *Rattus norvegicus* was the most susceptible rodent species to this parasitic infection followed by *Rattus rattus* and *Mus musculus* (Table 1). It was observed that this parasitic infection varied significantly between the sampling sites for *Rattus norvegicus* as well as for *Rattus rattus* but remained unaffected for *Mus musculus* (Table 2). We also observed that *Toxoplasma gondii* infection was not associated with the particular sex of all four rodent species enrolled in this study (Table 3). Our results are in agreement with a recent study from Pakistan where Rizwan et al.^8^ has reported that *Toxoplasma gondii* infection rates were significantly higher in *Rattus norvegicus* than in *Rattus rattus*. Similar trend has been reported from the other parts of the world as well. Kalmár et al.^19^ had also reported significant variation in *Toxoplasma gondii* infection among the nine rodent species enrolled from Romania. Rizwan et al.^8^ reported that rodent species, gender, location, season and habitat type were not associated with *Toxoplasma gondii* prevalence in wild rodents from Sahiwal division in Pakistan. Similar observations were documented by Kalmár et al.^19^ from Romania and Mikhail et al.^21^ from Egypt. On the other hand, Hosseini et al.^5^ had reported that *Toxoplasma gondii* infection was not restricted to a particular sex of rat but adult rats and those captured from rural areas were more prone to this parasitic infection than juvenile rats and those captured from urban areas in northern Iran. Nazari et al.^16^ had also reported that *Toxoplasma gondii* infection was not limited to a particular rodent species in Iran but males had higher rate of infection than females. Normaznah et al.^20^ had reported a significant variation in *Toxoplasma gondii* prevalence in rodent species that were trapped from various locations in Malaysia. These contrasting findings underscore the complexity of factors influencing *Toxoplasma gondii* infection dynamics among rodent species including geographical location, age and sex necessitating further research for a better understanding of these patterns.

Clinical pathology evaluation is common in rodents during the studies where effect of parasites or toxicants is to be determined. These analyses also help to identify disease in specific target organ as well as provide a general health profile of the individual animal^36^. Complete blood count analysis is among the most commonly used clinical pathology tools despite of the fact that acquisition and analysis of blood samples from rodents’ mice is problematic as their body size are not large and small sample volumes are usually obtained from a single animal^37^. Analysis of our complete blood count data revealed that *Toxoplasma gondii* infection had disturbed the Lymphocyte count, mean corpuscular volume, mean corpuscular hemoglobin concentration and mean platelet volume in *Rattus norvegicus* and *Mus musculus* but the overall blood profile of *Rattus rattus* remained unaffected when compared between parasite positive and negative animals (Table 4). The decreased lymphocyte count observed in this study is probably due to deficient regulation of inflammatory response due to *Toxoplasma gondii* mediated infection which is reported to be capable of tissue damage in inflammatory response^38^. The decreased mean platelet volume observed in infected rodents during this study is in line with the findings of by El-Henawy et al.^39^ as they had reported a significant decrease in platelet counts and associated parameters in *Toxoplasma gondii* infected human subjects. The disturbed hemoglobin associated parameters in Toxoplasma gondii infected rodents observed in this investigation are confirming the finding of Wang et al. ^40^ who had reported that *Toxoplasma gondii*-infected mice exhibited anemia due to a decrease in both erythropoiesis and survival time of red blood cells in the circulation. In a recent report, Ode et al.^15^ have documented a significant decrease in platelet count while an increased granulocyte: lymphocyte ratio and increased lymphocyte: monocyte ratio in *Toxoplasma gondii* infected wild rats (*Zyzomys pedunculatus*) from Nigeria as compared to uninfected animals. Similarly, Zoghroban et al.^41^ had also observed significant differences in the neutrophil, lymphocytes, monocyte, eosinophil and red blood cell counts in Swiss albino mice that were experimentally infected with *Toxoplasma gondii* as compared to uninfected animals. As several rodent species are preferred animal models, a few studies has reported the effect of *Toxoplasma gondii* infection on their complete blood count under laboratory conditions but there is no such data available in literature from wild rodents. Hence, the data generated in this study is adding to the existing knowledge regarding the effect of this one of the prevalent protozoan parasites on the blood cell count and associated parameters of three Pakistani wild rodent species.

Parasites are among the biotic factors of the environment that regulates the wild animals and they are known to affect their host by several ways. For example, they may reduce host’s growth, prevent them from reproducing or change their behavior^42^. Parasitic activities result in the stimulation of the host’s immune system that led to the generation of toxic oxidants that should counteracted by the antioxidant system of the host otherwise a state of oxidative stress may occur^43^. These oxidants are either produced due to increased metabolic process to combat the infection or they are generated by the cytotoxic cells to kill the pathogens^44^. Parasites themselves are known to release oxidants by the degradation of their own metabolic products. These toxic oxidants have the potential to damage host tissues. These oxidative damages may lead to degenerative pathologies that shorten the lifespan of host^45^. Defenses against reactive ions mediated damage include the enzymes catalase and as well as superoxide dismutase. Superoxide dismutases are metalloenzymes that are found in all life forms and are considered as first line of defense against the oxidative stress as they catalyze the dismutation of superoxide anion free radical (O_2_^−^) into molecular oxygen and hydrogen peroxide (H_2_O_2_)^46^. Catalase is an extremely efficient enzyme with the ability to break down millions of H_2_O_2_ molecules per second and convert them into water and oxygen in an energy-efficient manner ^47^. The free radicals generated in a cell following the parasitic activity attacks the lipids in the membranes, especially polyunsaturated fatty acids, and convert them into peroxide and hydroperoxide that trigger further reactions that lead to DNA and protein modifications: the process known as lipid peroxidation^48^. One of the end products of lipid peroxidation is malondialdehyde, an extremely toxic compound, which is usually determined to estimate the catastrophy of the membrane^49^.

*Toxoplasma gondii* is known to invade a number of the tissues of their diverse hosts including wild rodents but little has been reported regarding the response of host tissues to this infection through their antioxidant system^5,50^. Hence, we have compared the Superoxide dismutase, catalase (antioxidants) and Malondialdehyde (marker of oxidative stress) levels between *Toxoplasma gondii* infected and uninfected rodents. Analysis of the results indicated that the three rodent species should different response to *Toxoplasma gondii* infection as far as the markers of oxidative strength was concerned (Tables 5, 6 and 7). Like complete blood count analysis, the antioxidant profile from the vital organs of *Rattus rattus* remained unaffected when compared between *Toxoplasma gondii* infected and uninfected animals (Table 7). Infected *Mus musculus* had elevated malondialdehyde in kidney and liver while kidney of infected *Rattus norvegicus* had also significantly higher levels of this indicator of lipid peroxidation indication sever membrane damage following parasitic infection. Kidney and heart of *Toxoplasma gondii* infected *Mus musculus* and lungs of infected *Rattus norvegicus* had disturbed catalase concentrations that may lead to the accumulation of H_2_O_2_ in these cells that can trigger catastrophy leading to disturbed physiology. Prior to this data, there is only one report available in literature where et al. Nazarlu et al.^51^ had reported a significant decrease in superoxide dismutase, catalase, glutathione and total antioxidant capacity while the concentration of malondialdehyde was increased in the testes of *Toxoplasma gondii* infected male. Our results are indicating the markers if oxidative stress must be studies in all the tissues of rodents to get a better understanding of the pathophysiology of toxoplasmosis.

In conclusion, we are reporting a relatively higher *Toxoplasma gondii* infection in four Pakistani rodent species. Parasite prevalence varied between the rodent species and *Rattus norvegicus* had the highest rate of infection where more than one species members were found infected. *Toxoplasma gondii* infection disturbed the red, white blood cells and platelet associated parameters as well as markers of oxidative stress in liver, kidney and lungs of infected rodents. We recommend that similar and large-scale studies to be conducted in various geo-climatic regions of Pakistan to report the prevalence of *Toxoplasma gondii* in rodents for their effective control in order to prevent the zoonotic spread of this parasite through rodents.

## Data Availability

The datasets generated and/or analysed during the current study are available in the GenBank repository, with Accession numbers OR797081, OR797082, OR797083, OR797084 (https://www.ncbi.nlm.nih.gov/nuccore/OR797082.1https://www.ncbi.nlm.nih.gov/nuccore/OR797082.1https://www.ncbi.nlm.nih.gov/nuccore/OR797084.1 https://www.ncbi.nlm.nih.gov/nuccore/OR797083.1.

## Declarations

### ARRIVE guidelines

All the methods were performed in accordance with ARRIVE guidelines laws and regulations.
